# Comparative Analysis of Adaptation Behaviors of Different Types of Drivers to Steer-by-Wire Systems

**DOI:** 10.3390/s24175562

**Published:** 2024-08-28

**Authors:** Chen Chen, Liqiang Jin, Hongyu Zheng, Changfu Zong

**Affiliations:** The National Key Laboratory of Automotive Chassis Integration and Bionics, Jilin University, Changchun 130022, China; chen_chen22@mails.jlu.edu.cn (C.C.); jinlq@jlu.edu.cn (L.J.); zongcf@jlu.edu.cn (C.Z.)

**Keywords:** adaptation behaviors, driver adaptability, steer-by-wire systems, novice drivers, experienced drivers

## Abstract

As one of the advanced automotive chassis technologies, the steer-by-wire system offers a high level of precision, responsiveness, and controllability in the driving experience. It can also adjust and optimize parameters to adapt to the preferences of different drivers. However, when faced with the steer-by-wire system, both experienced drivers and novice drivers are in the novice stage, exhibiting learning or adaptation behaviors when using this steering system. In this paper, a small-scale pilot evaluation was conducted by means of a questionnaire survey and driving-simulator experiment, and the learning behavior and adaptability of four experienced and four novice drivers to the steer-by-wire system were analyzed when using the traditional steering system. The study found that experienced drivers show significant changes in their adaptation to the steering system, mainly due to their habitual driving with traditional steering systems. In contrast, novice drivers show no significant changes in their adaptation to the steering system, which is attributed to their lack of driving experience and skills, resulting in less sensitivity to changes in the steering system. Additionally, the study found that novice drivers under the steer-by-wire system grasp control over speed and steering-wheel angle more quickly. This research provides a reference for improving drivers’ learning and adaptation abilities to the steer-by-wire system and optimizing the design of the steer-by-wire system.

## 1. Introduction

With the development of automotive intelligence, traditional mechanical steering systems are gradually facing limitations and challenges, such as certain limitations on feedback accuracy, sensitivity, and maneuverability. In contrast, steer-by-wire systems employ electronic control technology to achieve steering operations through electronic signals, greatly enhancing the precision and flexibility of the steering system [1,2]. Unlike traditional systems, steer-by-wire systems eliminate the mechanical and hydraulic linkages between the steering wheel and the wheels, replacing them with electronic sensors, actuators, and control units. This allows for more precise and immediate responses to driver inputs, as well as the integration of advanced driver-assistance systems and autonomous driving technologies. Moreover, steer-by-wire systems can be adjusted according to driving conditions, vehicle status, and driver types, thereby improving driving safety, stability, and comfort [3,4]. Additionally, these systems offer the potential for more innovative vehicle designs, as they remove the need for mechanical steering columns, providing more interior space and flexibility in vehicle architecture. Steer-by-wire systems offer many advantages in autonomous vehicles, but compared to traditional mechanical steering systems, they exhibit significant differences in control methods and characteristics [5]. Therefore, drivers may encounter adaptability issues when initially using steer-by-wire systems, especially those accustomed to traditional steering systems who may need to alter their operating behaviors to relearn or adapt to steer-by-wire systems.

Currently, most scholars are devoted to researching the adaptability of vehicle systems, aiming to enable vehicles to autonomously perceive surrounding environments and driver intentions, make intelligent decisions, and execute the corresponding actions, thereby improving vehicle safety, efficiency, and comfort [6,7]. However, as drivers accumulate driving mileage, they also exhibit adaptive behaviors towards vehicle systems, thereby changing their operating behaviors to adapt to the systems. So far, some countries have initiated assessments of driver-behavior adaptability [8,9]. Some scholars research driver-behavior changes and design vehicle systems [10,11]. There are also some scholars who have researched the adaptability of drivers to new vehicle technologies, such as driver-assistance systems [12,13,14]. Additionally, in-depth studies have been conducted on driver interaction with automated vehicles [15,16]. Driver adaptability is affected not only by age and gender but also by personality traits, driving style, driving skills, and sensation-seeking tendencies [17,18]. At the same time, the surrounding environment, traffic conditions, and other external factors also impact it [19,20,21]. Research on driver-behavior adaptability is helpful for better understanding the adaptation processes and characteristics of drivers in different driving scenarios and providing guidance for designing roads, vehicle control systems, and other elements that meet driver needs [22,23,24]. Furthermore, research on driver-behavior adaptability can help reduce driver discomfort with new technologies and increase driver trust and satisfaction with vehicle control systems [25].

Research on driver-behavior adaptation mainly focuses on traditional vehicles, automated vehicles, driver-assistance systems, etc., with a particular emphasis on studies involving elderly drivers [26,27,28]. However, the differences in behavior adaptability among different types of drivers have not been considered. Therefore, this paper researches the behavior adaptation changes and influencing factors of different types of drivers based on the steer-by-wire system. Faced with the steer-by-wire system, both experienced drivers accustomed to traditional steering systems and less-experienced drivers may feel unfamiliar when encountering and using this new type of steering system. Experienced drivers with traditional steering systems are familiar with its operation and exhibit certain habitual behaviors. In contrast, novice drivers with traditional steering systems may lack safety awareness and driving experience and may not be as adept at controlling the vehicle or responding to complex traffic environments as experienced drivers with traditional steering systems [29,30,31]. Therefore, it is unknown which type of driver can accept and adapt to the steer-by-wire system more quickly and effectively, and the time and influencing factors for drivers to adapt to the steer-by-wire system are also uncertain. Thus, based on the steer-by-wire system, this paper conducts research on the adaptive behavior and learning of different types of drivers towards this system, aiming to investigate the behavior adaptation situations and primary influencing factors caused by the steer-by-wire system among different types of drivers.

The experiment analyzed in this paper is about the adaptability of experienced and novice drivers to the steering-by-wire system and traditional steering system. Four experienced drivers and four novices for each steering system were selected. The data collection methods included questionnaire surveys and driving-simulator tests to gather basic information, driving styles, and driving data under different steering systems. Subsequently, the data from both types of drivers under steering-by-wire and traditional steering systems were processed to compare and analyze differences in driving characteristics, behavioral changes, and adaptability. The main contributions of this article are as follows:

1. Comparative analysis of driving behaviors: by contrasting the behaviors of experienced and novice drivers under traditional and steer-by-wire steering systems, the article reveals distinct characteristics in drivers’ acceptance and adaptation processes to the steer-by-wire steering systems;
2. Exploration of factors influencing adaptability: The article investigates the impact of factors such as driving experience, skills, and styles on drivers’ adaptability to steer-by-wire systems. Through the collection and analysis of experimental data, it examines how different types of drivers adapt to changes in steering systems, offering new insights into understanding driver-behavioral adaptability;
3. Observation and evaluation of novice drivers’ growth behavior: The article observes and evaluates the growth behavior of novice drivers under different steering systems. Analyzing their driving behaviors under traditional and steer-by-wire systems provides deeper insights into their progression from initial exposure to gradual mastery of new technology. This contributes to designing steer-by-wire systems tailored to novice drivers’ needs.

The structure of this paper is as follows. Section 2 is for experiment data collection, conducting experiments on experienced drivers and novice drivers driving traditional steering systems and steer-by-wire systems. Section 3 is for analyzing the experimental data results, processing the data of experienced drivers and novice drivers driving traditional steering systems and steer-by-wire systems. Section 4 is for analyzing the behavioral changes and adaptation situations of different types of drivers under steer-by-wire systems. Section 5 is for the conclusion and outlook of this paper.

## 2. Method

### 2.1. Participants

This paper proposes two levels of drivers, A-level drivers and B-level drivers, based on driving simulators equipped with different steering systems. A-level drivers are defined as drivers with less than one year of driving experience and less than five thousand kilometers of driving mileage or drivers without experience in driving vehicles equipped with traditional steering systems other than to obtain a driver’s license, and they are required to have no experience in driving vehicles equipped with steer-by-wire systems and no experience in using driving simulators. B-level drivers are defined as drivers with more than three years of driving experience and over twenty thousand kilometers of driving mileage, who have driving experience every week, and they are required to have no experience in driving vehicles equipped with steer-by-wire systems and no experience in using driving simulators.

A total of 14 drivers were recruited through online announcements and were compensated for their participation. Among the 14 drivers, there were 7 A-level drivers and 7 B-level drivers. The age range was between 20 and 50 years old. The driving experience of the B-level drivers ranged from 5 to 20 years, and their driving mileage ranged from 20,000 km to 100,000 km. All participants in the experiment were in good health, and their vision was either normal or corrected to normal. Before participating in the study, the participants were informed that they could withdraw at any time for any reason, and all participants provided informed consent.

### 2.2. Experimental Design

(1) Questionnaire Design

A questionnaire was developed for this experiment and divided into two parts. The first part was designed to collect basic information about the drivers, including driving style, driving skills, and other related information. The second part aimed to gather information about the drivers’ experiences and feelings when driving vehicles equipped with the steer-by-wire system. The questionnaire was administered in two stages. The drivers completed the first part before the experiment began and the second part after the experiment concluded.

The first part of the questionnaire included a series of 5-point Likert scale questions, where 1 indicated “strongly disagree” and 5 indicated “strongly agree.” The second part comprised five questions primarily focused on collecting drivers’ experiences, perceptions, and levels of acceptance and trust in vehicles equipped with the steer-by-wire system;

(2) Driving-Simulator Experiment Design

Research shows that driving simulators are highly valid and reliable for studying driving behaviors and responses. Moreover, driving simulators can simulate real driving scenarios in a safe, controlled, and reproducible environment. They allow for precise control of the experimental conditions, enabling the capture of driver-behavior data under different steering systems, particularly ensuring the safety of novice drivers during experiments [32,33]. The study employed a static driving simulator, which can meet the requirements of driver-steering data collection. Although the driving simulator differs from the real world in terms of dynamic driving experiences like acceleration, deceleration, and road turbulence, it adeptly simulates force feedback through control devices like the steering wheel and pedal assembly. Furthermore, it presents a comprehensive driving environment via its display system, catering to the driver’s visual needs. Research has corroborated the effectiveness and reliability of fixed-base driving simulators for evaluating driving behavior [34]. Specifically, in experiments related to steering systems, these simulators are effective at simulating driving environments and precisely controlling variables [35]. In conclusion, the driving simulator utilized in this study effectively fulfills the specific testing requirements outlined in this paper.

**Figure 1:**
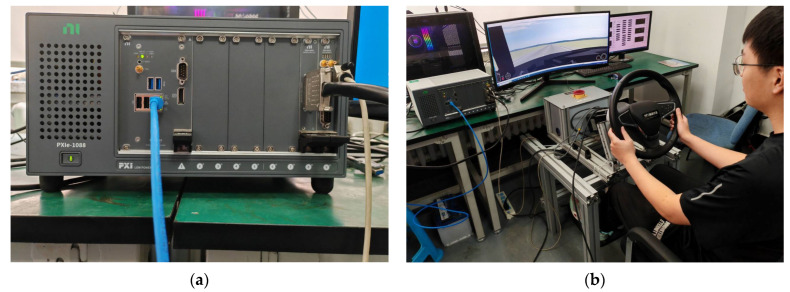
Experimental equipment introduction. (**a**) The bottom machine in the driving simulator; (**b**) driving-simulator platform.

The driving simulator consists of a steering wheel, pedal assembly, and seat. The vehicle selected is a C-class hatchback (wheelbase = 2910 mm, sprung mass = 1270 kg, width for animator = 2082 mm, height for animator = 1450 mm, and height of center of mass = 540 mm), operating in automatic transmission mode, eliminating the need for participants to use a clutch. The simulator is equipped with an LCD display, presenting road and surrounding conditions to the driver, as well as displaying dashboard information, which is shown in Figure 1. During the experiment, the accelerator pedal, brake pedal, and steering wheel are powered and function normally. The participants complete designated driving tasks by operating these three devices.

**Figure 2:**
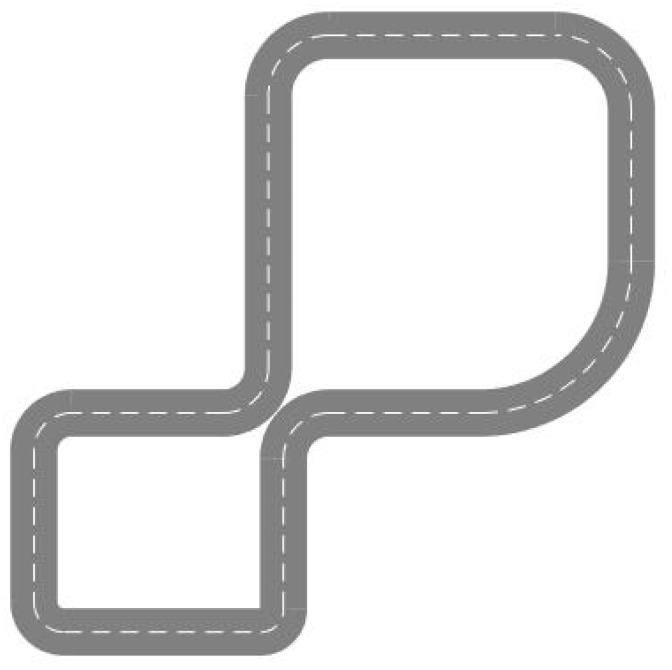
Experimental Path Diagram.

Since the experiment primarily aims to validate the drivers’ adaptability to the steer-by-wire system, it requires comparing and analyzing the relevant data during steering maneuvers. Therefore, the experimental path is designed with multiple curved-road scenarios. There are a total of eight curves, with six left curves and two right curves, considering the difficulty of right turns, which are challenging for A-level drivers to handle. Each curve has a different radius, 50, 40, 30, 25, 20, 15, and 30 m. In turn, each is connected by a straight road, as illustrated in Figure 2. The experimental road conditions consist of two one-way lanes, each 4 m wide, spanning a length of 1.45 km, without the ups and downs of the road. There are only test vehicles on the road and no other vehicles or pedestrians.

### 2.3. Produce

Before experimenting, the drivers are required to fill out an informed consent form and the first part of the questionnaire. They also need to complete a questionnaire about their emotional state to ensure that their emotional state is normal before proceeding with the subsequent driving-simulator experiment.

Before the formal experiment, a pre-experiment session is conducted, during which drivers have 10 to 20 min to familiarize themselves with the use of the driving simulator. During this time, the driver gains acceleration and brake pedal and steering-wheel control, can complete full-speed steering, and can correctly use the driving simulator to complete the driving task on the specified road. After the pre-experiment session, the drivers have a 10-min break before the formal experiment begins.

The formal experiment is divided into two parts. One part involves the traditional steering system experiment, and the other part involves the steer-by-wire system experiment. Among them, the traditional steering system is controlled by a fixed transmission ratio of 15, while the transmission ratio scheme of the steer-by-wire steering system adopts a variable angle transmission ratio scheme based on a constant yaw angle speed gain [36]. The fixed transmission ratio in the traditional steering system means that the ratio of the steering-wheel angle to the steering wheel is fixed.

**Figure 3:**
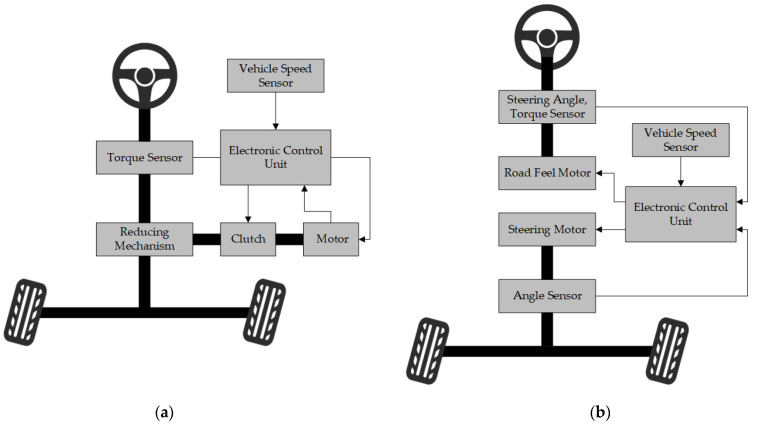
Structure comparison between steering-by-wire system and traditional steering system. (**a**) Traditional steering system; (**b**) steer-by-wire system.

Unlike traditional steering systems, steer-by-wire systems eliminate the mechanical and hydraulic connections between the steering wheel and the wheels, replacing them with electronic sensors, actuators, and control units. The steer-by-wire system dynamically adjusts the transmission ratio according to different driving conditions, such as speed and steering-wheel angle, to maintain the best handling performance and vehicle stability. In addition, the steer-by-wire system incorporates road-sensation simulation, mimicking the resistance and feel of traditional mechanical steering systems to provide haptic feedback to the driver. The specific structural differences between the two steering systems are shown in Figure 3.

The experimental conditions are consistent for both parts, with only the different steering systems. Before the formal experimental data collection, we conducted experiments on the influence of different steering system test sequences on the driver status and found that the influence of varying steering system test sequences on the driver status was not significant. In addition, since the purpose of this paper is mainly to observe the adaptability changes of different types of drivers to steer-by-wire systems, this paper first conducted the steer-by-wire system test. After that, the traditional steering system was tested. The drivers were not informed about which steering system they were using during the experiment.

There are no strict requirements for the driving speed of the drivers during the experiment, but the drivers are required to stay in the right lane. The experiment collects driving parameters such as longitudinal speed, longitudinal acceleration, lateral speed, steering-wheel angle, and steering-wheel angle velocity to analyze the driver’s adaptability and learning behavior. Each driver conducts 15 drives with each steering system, divided into groups of five. After completing each group of experiments, drivers have a 10-min break before continuing with the next set of experiments.

During the experiment, if the vehicle deviates from the road, the trial is considered invalid, and it needs to be restarted to avoid unnecessary experiments and data collection, thereby improving the efficiency of data collection. After the experiment concludes, drivers are required to fill out a questionnaire about their experience driving the steer-by-wire system vehicle.

### 2.4. Data Collection and Statistical Analysis

The driver’s operational behavior data (steering-wheel angle, steering-wheel angle velocity, throttle and brake pedal stroke, etc.) and vehicle motion data (longitudinal speed, lateral speed, vehicle position, etc.) are collected at a frequency of 500 Hz.

Based on the driver’s throttle-pedal data, preliminary processing is conducted to exclude data points where the driver was not engaged in driving tasks. Driving data during cornering is extracted based on the vehicle’s longitudinal and lateral coordinates for subsequent driver analysis.

By extracting and analyzing the feature parameters, it is found that there are obvious differences between different drivers regarding speed and steering-wheel angle control. Therefore, the following data are selected for driver analysis: average longitudinal vehicle speed, longitudinal speed standard deviation, maximum steering-wheel angle, steering-wheel angle standard deviation, and driving completion time, as shown in Table 1.

In this paper, the significance of the characteristic parameters between A-level and B-level drivers is examined. Initially, Levene’s test was employed to assess the homogeneity of variances between the two groups of data. However, it was determined that the data did not meet the necessary criterion for variance homogeneity. Consequently, the Mann–Whitney U test, from the realm of non-parametric tests, was chosen to analyze the significant differences between the two groups. This method is advantageous in that it does not necessitate normal distribution of the data and can effectively test for significant differences among distinct datasets.

The results revealed significant differences in several key parameters, including average speed (Z = −14.04, *p* < 0.05), maximum speed (Z = −12.19, *p* < 0.05), maximum steering angle (Z = −5.78, *p* < 0.05), minimum steering-wheel angle (Z = −2.72, *p* < 0.05), and maximum absolute steering-wheel angle (Z = −5.49, *p* < 0.05), among others. Given these statistically significant disparities, the subsequent analysis in this paper focuses specifically on the speed and steering-wheel angle data. This targeted approach aims to provide deeper insights into the performance differences between the two steering systems under investigation.

## 3. Result

### 3.1. Driver Questionnaire Survey Results

By summarizing and consolidating the questionnaire survey results, we obtained the driving styles, driving skill levels, understanding level of the steer-by-wire system, trust level, acceptance level, and driving experiences of the eight drivers, as shown in Table 2. It can be seen that some drivers have knowledge about the steer-by-wire system. Most of them hold a trusting and accepting attitude towards it, and their driving experiences with the steer-by-wire system are generally positive. B-level drivers can feel the differences between the steer-by-wire system and the traditional steering system, while A-level drivers are less sensitive to the changes in the steering system.

### 3.2. Analysis of Driving Data Variation for Different Types of Drivers

#### 3.2.1. Speed Variation Curves

**Figure 4:**
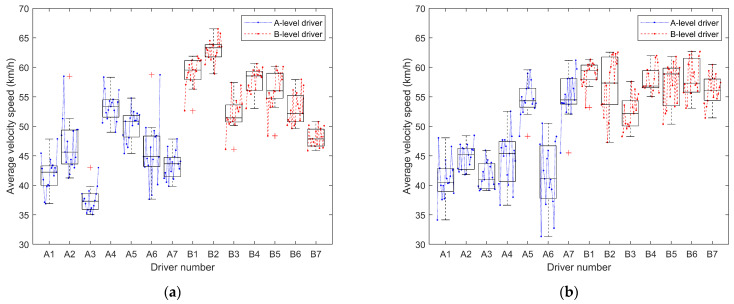
Variation of average longitudinal vehicle speed with driving times. (**a**) Steering-by-wire system; (**b**) traditional steering system.

**Figure 5:**
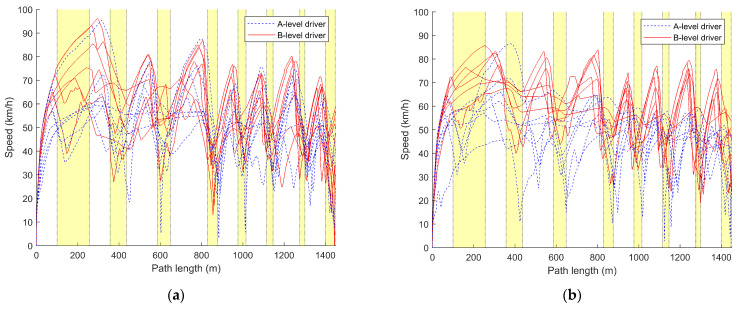
Comparison curve of speed variation with distance during the driver’s initial drive. (**a**) Steering-by-wire system; (**b**) traditional steering system. The yellow part of the figures shows the position of the curve in the driving condition. The same goes for the figures below.

**Figure 6:**
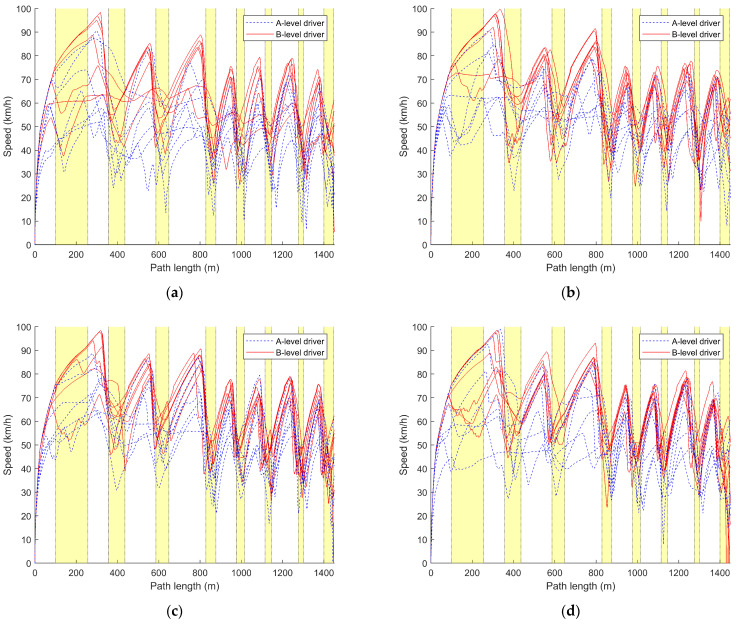
Comparison curve of speed variation with distance during the 8th and 15th driving times. (**a**) The 8th drive with steer-by-wire system; (**b**) the 8th drive with traditional steering system; (**c**) the 15th drive with steer-by-wire system; (**d**) the 15h drive with traditional steering system.

In this section, the average driving speed over fifteen driving sessions for each driver is calculated, analyzed, and plotted, as shown in Table 3 and Table 4 and Figure 4. Additionally, the speed variation during the first, eighth, and fifteenth driving sessions for each driver is plotted against the distance traveled, which is illustrated in Figure 5 and Figure 6.

In this section, the average speed parameters of A-level and B-level drivers under the steer-by-wire system and traditional steering system are tested for significant differences. For the data of A-level drivers driving two different steering systems, Levene’s test of variance homogeneity is performed first. The calculation results found that the homogeneity requirements were met. So, one-way analysis of variance (ANOVA) was selected for the significance difference test, and the calculated results (F = 6.11, *p* < 0.05) showed significant differences. And for the data of B-level drivers driving two different steering systems, Levene’s test of variance homogeneity is also performed first. The calculation results found that the homogeneity requirements were met. So, one-way ANOVA was selected for the significance difference test, and the calculated results (F = 7.404, *p* < 0.05) showed significant differences.

Combining Table 3 and Table 4, it can be observed that the average vehicle speed for A-level drivers (M_A_SBW = 45.519, Med_A_SBW = 44.749, M_A_nSBW = 46.200, Med_A_nSBW = 45.373) is significantly lower than that of B-level drivers (M_B_SBW = 55.594, Med_B_SBW = 56.031, M_B_nSBW = 56.789, Med_B_nSBW = 56.986). Additionally, the average vehicle speed data for A-level drivers (R_A_SBW = 23.674, SD_A_SBW = 5.866, R_A_nSBW = 29.864, SD_A_nSBW = 6.740) exhibits greater dispersion compared to that of B-level drivers (R_B_SBW = 20.642, SD_B_SBW = 5.205, R_B_nSBW = 15.394, SD_B_nSBW = 3.695), indicating larger variations. These findings suggest that A-level drivers typically drive at lower speeds, with greater fluctuations, while B-level drivers drive at higher and more consistent speeds. The line graphs in Figure 4 depict that, initially, A-level drivers have lower average speeds, which increase with the number of trials. However, some A-level drivers consistently maintain lower average speeds. It can also be observed that A-level drivers have less control over their speeds, with larger fluctuations compared to B-level drivers, whose average speeds remain relatively stable within a certain range after the initial trials.

Comparing Table 3 and Table 4 along with Figure 4a,b, it can be observed that both the traditional steering system and the steer-by-wire system have a relatively minor impact on the driving speed of A and B-level drivers. For A-level drivers, the control of speed is better under the steer-by-wire system (M_A_SBW = 45.519, R_A_SBW = 23.674, SD_A_SBW = 5.866) compared to the traditional steering system (M_A_nSBW = 46.200, R_A_nSBW = 29.864, SD_A_nSBW = 6.740). However, the speed under the steer-by-wire system is lower than that under the traditional steering system. For B-level drivers, the speed control under the steer-by-wire system (M_B_SBW = 55.594, R_B_SBW = 20.642, SD_B_SBW = 5.205) is poorer compared to the traditional steering system (M_B_nSBW = 56.789, R_B_nSBW = 15.394, SD_B_nSBW = 3.695), but B-level drivers have a higher average speed under the steer-by-wire system.

From the speed–distance curves of the drivers’ initial driving in Figure 5, it can be observed that the speed-control ability of A-level drivers is significantly lower than that of B-level drivers. This is mainly reflected in the frequent and abrupt corrections in speed, as well as instances of sustained low-speed driving, failure to decelerate in corners, and inappropriate acceleration and deceleration. B-level drivers exhibit better speed control, smoothly adjusting speed when entering and exiting corners. Overall, their speed is higher than that of A-level drivers. However, due to their first-time driving experience, they may not be familiar with the conditions and may need to make speed adjustments in corners.

Comparing the curves labeled (a) and (b) in Figure 5, it can be seen that, under the traditional steering system, B-level drivers generally exhibit better speed control than under the steer-by-wire system. However, their speed is lower compared to the steer-by-wire system, and there is significant speed fluctuation in corners. For A-level drivers, under the steer-by-wire system, the speed fluctuation during cornering is smaller compared to the traditional steering system, but there are more speed corrections compared to the traditional steering system.

Comparing Figure 5 with Figure 6 reveals that A-level drivers gradually improve their speed control with increased driving experience. They demonstrate relatively appropriate acceleration and deceleration during turns, although frequent corrections are still required, and their speeds remain lower compared to B-level drivers. Moreover, it can be observed that A-level drivers exhibit faster speed control under the steer-by-wire system, approaching the proficiency of B-level drivers by the 8th driving, where they appropriately decelerate at turns. In contrast, under the traditional steering system, A-level drivers begin to approach the proficiency of B-level drivers after the 15th driving.

While B-level drivers initially struggle with speed control during their first driving, their control gradually improves with increased driving experience. They demonstrate smoother acceleration and deceleration during turns, with minimal corrections to speed. B-level drivers excel at speed control under the traditional steering system, with reduced speed fluctuations as attempts increase. By the 15th driving, regardless of whether it is under the steer-by-wire system or the conventional steering system, B-level drivers’ speed control is nearly identical. However, under the traditional steering system, their speed during turns is higher, with smaller speed fluctuations.

From the above figures and tables, it can be seen that A-level drivers have lower average speeds, more pronounced speed variations, and less stable control. Their speed control is slightly better under the steer-by-wire system compared to the traditional steering system. B-level drivers maintain slightly lower average speeds under the traditional steering system, with more stable speed data. However, there are more corrections under the steer-by-wire system. Additionally, B-level drivers outperform A-level drivers in speed control under both steering systems. While different steering system types indeed influence drivers’ speed control, improving speed control remains a focal concern for A-level drivers regardless of the steering system used.

#### 3.2.2. Steering-Wheel Angle Variation Curve

**Figure 7:**
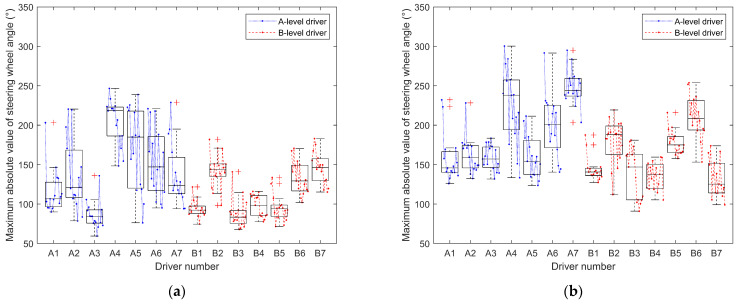
Variation of average maximum absolute steering-wheel angle with driving times. (**a**) Steering-by-wire system; (**b**) traditional steering system.

**Figure 8:**
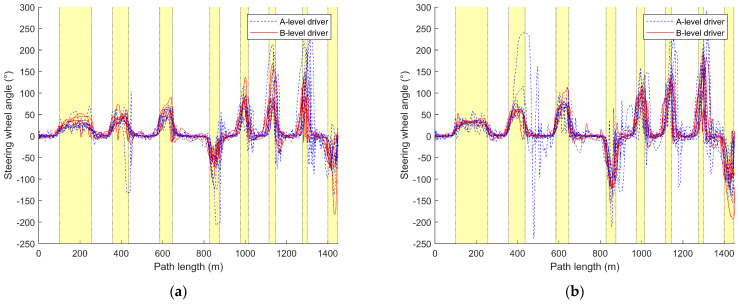
Comparison curve of steering-wheel angle with distance during the driver’s initial drive. (**a**) Steering-by-wire system; (**b**) traditional steering system.

**Figure 9:**
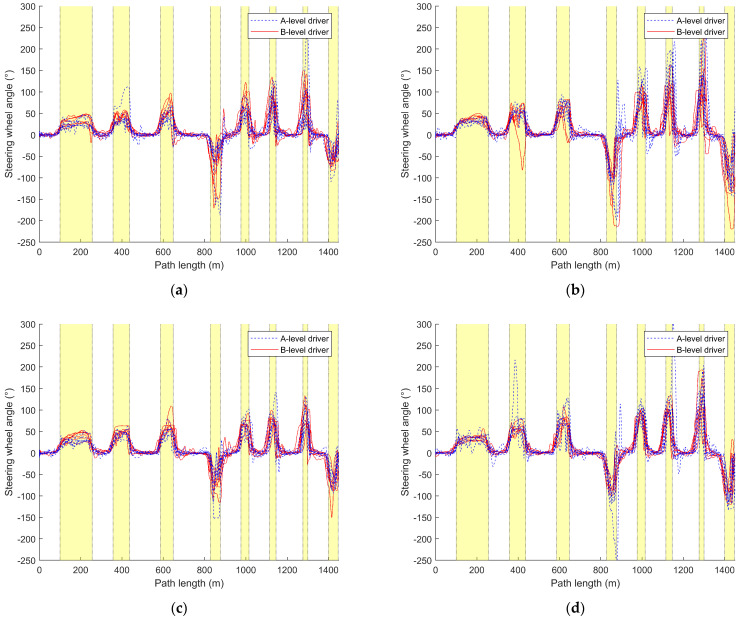
Comparison curve of steering-wheel angle with distance during the 8th and 15th driving times. (**a**) The 8th drive with steer-by-wire system; (**b**) the 8th drive with traditional steering system; (**c**) the 15th drive with steer-by-wire system; (**d**) the 15h drive with traditional steering system.

This section calculates and analyzes the maximum absolute value of the steering-wheel angle for each driver’s fifteen drives, as shown in Table 5 and Table 6 and illustrated in Figure 7. Furthermore, it plots the variation of the steering-wheel angle with distance for the first, eighth, and fifteenth drives of each driver, as depicted in Figure 8 and Figure 9.

In this section, the average maximum absolute steering-wheel angle parameters of A-level and B-level drivers under the steer-by-wire system and traditional steering system are tested for significant differences. For the data of A-level and B-level drivers driving two different steering systems, Levene’s test of variance homogeneity is performed first. The calculation results found that the homogeneity requirements were met, so one-way ANOVA was selected for the significance difference test. The calculated results of A-level drivers (F = 42.05, *p* < 0.05) and B-level drivers (F = 101.48, *p* < 0.05) both showed significant differences.

Combining Table 5 and Table 6, it can be observed that the maximum absolute steering-wheel angle values for A-level drivers (M_A_SBW = 143.56, Med_A_SBW = 129.15, M_A_nSBW = 187.26, Med_A_nSBW = 173.08) are significantly higher than those for B-level drivers (M_B_SBW = 113.83, Med_B_SBW = 108.94, M_B_nSBW = 159.77, Med_B_nSBW = 158.49). This indicates that A-level drivers tend to apply larger steering-wheel angles during driving, possibly due to their frequent steering maneuvers. Additionally, the data for the maximum absolute steering-wheel angle values are more dispersed for A-level drivers (R_A_SBW = 187.17, SD_A_SBW = 51.15, R_A_nSBW = 176.72, SD_A_nSBW = 46.39) compared to B-level drivers (R_B_SBW = 115.32, SD_B_SBW = 29.58, R_B_nSBW = 162.89, SD_B_nSBW = 36.18), implying greater variability in steering-wheel usage among A-level drivers. From Figure 7, it is evident that A-level drivers exhibit poorer control over the steering-wheel angle compared to B-level drivers. As the number of driving instances increases, the maximum absolute steering-wheel angle values for A-level drivers gradually converge towards those of B-level drivers. B-level drivers maintain their maximum absolute steering-wheel angle values within a lower and more stable range compared to A-level drivers, and with an increase in the number of driving instances, these values gradually decrease.

Comparing Table 5 and Table 6, and Figure 7a,b, it can be observed that, regardless of whether it is A-level or B-level drivers, the maximum absolute steering-wheel angle values under the traditional steering system (M_A_nSBW = 187.26, Med_A_nSBW = 173.08, M_B_nSBW = 159.77, Med_B_nSBW = 158.49) are generally higher than those under the steer-by-wire system (M_A_SBW = 143.56, Med_A_SBW = 129.15, M_B_SBW = 113.83, Med_B_SBW = 108.94,). Additionally, under the traditional steering system, the maximum absolute steering-wheel angle values for A-level drivers are closer to those of B-level drivers. A-level drivers exhibit significant fluctuations in the maximum absolute steering-wheel angle values when driving with both the steer-by-wire system (R_A_SBW = 187.17, SD_A_SBW = 51.15) and the traditional steering system (R_A_nSBW = 176.72, SD_A_nSBW = 46.39). On the other hand, B-level drivers maintain relatively stable control over the steering-wheel angle when driving with the steer-by-wire system (R_B_SBW = 115.32, SD_B_SBW = 29.58), with the maximum absolute steering-wheel angle values gradually decreasing and stabilizing with an increase in the number of driving instances. However, when driving with the traditional steering system (R_B_nSBW = 162.89, SD_B_nSBW = 36.18), there is greater fluctuation in the maximum absolute steering-wheel angle values.

From Figure 8, it is evident that A-level drivers exhibit larger steering-wheel angles compared to B-level drivers, with more instances of both oversteering and understeering. They also struggle with timing the steering-wheel movements during turns and require more corrections to the steering-wheel angle, while B-level drivers also exhibit corrections during turns, albeit far fewer than A-level drivers.

Comparing (a) and (b) in Figure 8, it is evident that A-level drivers demonstrate better control of the steering-wheel angle during turns under the steer-by-wire system compared to the traditional steering system, especially for a smaller turn radius. Under the traditional steering system, as the turn radius decreases, A-level drivers exhibit poorer timing of steering-wheel movements during turns compared to those under the steer-by-wire system, requiring more corrections and experiencing increased instances of oversteering.

B-level drivers show superior control of the steering-wheel angle under the traditional steering system compared to the steer-by-wire system. However, under the steer-by-wire system, B-level drivers require more corrections to the steering-wheel angle, with slight instances of both oversteering and understeering. Nonetheless, under the SBW system, B-level drivers exhibit smaller steering-wheel angles, with minimal increases in steering-wheel angle as the turn radius decreases. In contrast, under the traditional steering system, both A-level and B-level drivers demonstrate larger steering-wheel angles overall compared to the steer-by-wire system, with the steering-wheel angle gradually increasing as the turn radius decreases. Regardless of driver level, traditional steering systems generally result in larger steering-wheel angles compared to steer-by-wire systems, and right turns are more prone to deviating from the expected steering-wheel angle than left turns.

Comparing Figure 8 and Figure 9, it can be observed that, as the number of driving sessions increases, A-level drivers gradually approach the level of steering angle control exhibited by B-level drivers, but they make more corrections to the steering angle during turns than B-level drivers. Initially, A-level drivers demonstrate better steering angle control under the steer-by-wire system compared to the traditional steering system, but later, their performance improves more under the traditional steering system. In the early stages of driving, A-level drivers under the steer-by-wire system approach the performance of B-level drivers more rapidly, reaching a similar level by the 8th driving. However, they make more corrections to the steering angle during turns. Under the traditional steering system, there remains a significant gap between A-level drivers and B-level drivers in terms of their ability to gauge steering timing and steering angle control. In the later stages of driving, A-level drivers’ timing and control of steering angle during turns under both traditional and steer-by-wire systems are closer to those of B-level drivers, but A-level drivers make fewer corrections to the steering angle during turns under the traditional steering system.

As the number of driving sessions increases, B-level drivers gradually reduce the steering angle and make fewer corrections, resulting in a smoother curve of steering angle variation over the course of the journey. Under the steer-by-wire system, B-level drivers also demonstrate a decrease in corrections to the steering angle during turns as the number of driving sessions increases, along with a reduction in instances of oversteering. However, under the traditional steering system, there is no significant change in the curve of steering angle variation with increased driving sessions for B-level drivers.

There are significant differences in steering angle between A-level and B-level drivers. The steering angle of A-level drivers is notably higher than that of B-level drivers, indicating that A-level drivers tend to apply larger steering angles, possibly due to the need for more frequent steering maneuvers. Additionally, the data for steering angle among A-level drivers are more dispersed, suggesting greater variability in their use of steering angle. Different steering systems also influence steering angle control among drivers. Regardless of A-level or B-level designation, steering angles are generally higher under the traditional steering system compared to the steer-by-wire system. B-level drivers show better adaptation to and faster learning of the steer-by-wire system compared to A-level drivers. The increase in driving sessions affects drivers’ control of the steering angle. While A-level drivers gradually approach the steering angle control of B-level drivers with increasing driving sessions, they still require more corrections.

### 3.3. Adaptation of Different Types of Drivers’ Behavior

Many scholars have extensively researched driver-behavior evaluation, encompassing objective evaluation methods for steering comfort, using vehicle state data evaluating driver performance and real-time evaluation systems for heavy-duty vehicle driver acceleration and braking behaviors [37,38,39]. In this paper, based on previous scholarly studies and the testing and analysis of the significant differences mentioned above, we have selected parameters such as speed and steering angle as the primary evaluation metrics. The scoring function employs the inverse tangent function, incorporating weighting factors, amplitude factors, and phase factors, to construct a formula that assesses the driver’s behavioral adaptability and quantifies their characteristic parameters.

According to the test conditions, the driver’s behavioral adaptability score is segmented into ten parts, with each part meticulously controlled to a maximum of ten points through the adjustment of various factors. This approach ultimately yields a comprehensive evaluation formula on a hundred-point scale. These ten sections encompass evaluations of eight corner scenarios, an overall assessment of driving performance, and an overall evaluation of the time taken to complete the test.

**Figure 10:**
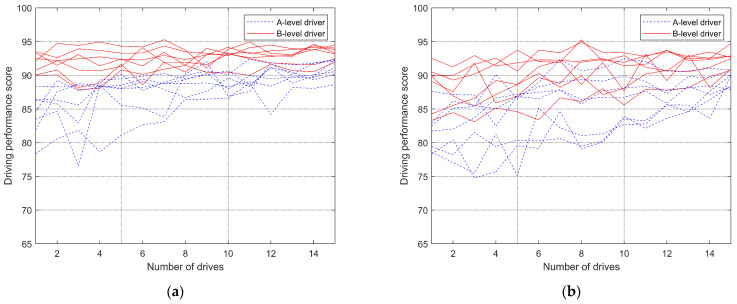
Score-change curve. (**a**) Steering-by-wire system; (**b**) traditional steering system.

The specific calculation formulas are detailed below, and the results presented in Figure 10 are derived by integrating the collected driver characteristic parameters. The specific value of the factor is selected and adjusted according to the data collected from the experiment.

The specific formula is shown below.

(1) $$G_{turn}=\alpha_{turn}-\frac{1}{\pi }\left[\begin{array}{c} \mu_{1}*\arctan\left(\beta_{1}*{Vx}_{std}+\varepsilon_{1}\right)+\mu_{2}*\arctan\left(\beta_{2}*{Vx}_{mean}+\varepsilon_{2}\right)\\ +\mu_{3}*\arctan\left(\beta_{3}*\theta_{std}+\varepsilon_{3}\right)+\mu_{4}*\arctan\left(\beta_{4}*\theta_{max}+\varepsilon_{4}\right) \end{array}\right]$$

where $G_{turn}$ is the curve score, $\alpha_{turn}$ is the curve base factor, $\mu_{1}$ is the weight factor of the standard deviation of curve speed, $\beta_{1}$ is the amplitude factor of the curve speed standard deviation, $\varepsilon_{1}$ is the phase factor of the curve speed standard deviation, ${Vx}_{std}$ is the standard deviation of the curve speed, $\mu_{2}$ is the weight factor of the average curve speed, $\beta_{2}$ is the amplitude factor of the average speed on curves, $\varepsilon_{2}$ is the phase factor of the average speed on curves, ${Vx}_{mean}$ is the average speed on curves, $\mu_{3}$ is the weight factor of the standard deviation of the steering-wheel corner on curves, $\beta_{3}$ is the amplitude factor of the standard deviation of the steering-wheel corner on curves, $\varepsilon_{3}$ is the phase factor of the standard deviation of the steering-wheel corner on curves, $\theta_{std}$ is the standard deviation of the steering-wheel angle on curves, $\mu_{4}$ is the weight factor of the maximum steering-wheel angle on curves, $\beta_{4}$ is the amplitude factor of the maximum steering-wheel angle on curves, $\varepsilon_{4}$ is the phase factor of the maximum steering-wheel angle in curves, and $\theta_{max}$ is the maximum steering-wheel angle in curves. In this paper, select $\alpha_{turn}=7,\;\mu_{1}=1,\;\beta_{1}=1.23,\;\varepsilon_{1}=-21.54,\;\mu_{2}=1,\;\beta_{2}=-0.31,\;\varepsilon_{2}=20,\;\mu_{3}=2,\;\beta_{3}=0.41,\;\varepsilon_{3}=-19.49,\;\mu_{4}=2,\;\beta_{4}=0.062,\;\varepsilon_{4}=-10.77.$

(2) $$G_{all}=\alpha_{all}-\frac{1}{\pi }\left[\mu_{5}*arctan\left(\beta_{5}*{Vxa}_{std}+\varepsilon_{5}\right)+\mu_{6}*arctan\left(\beta_{6}*{\theta a}_{std}+\varepsilon_{6}\right)+\mu_{7}*arctan\left(\beta_{7}*{\theta a}_{max}+\varepsilon_{7}\right)\right]$$

where $G_{all}$ is the score of the complete operating condition, $\alpha_{all}$ is the basic factor of complete operating condition, $\mu_{5}$ is the weight factor of the standard deviation of complete operating condition velocity, $\beta_{5}$ is the amplitude factor of complete operating condition velocity standard deviation, $\varepsilon_{5}$ is the phase factor of complete operating condition velocity standard deviation, ${Vxa}_{std}$ is the standard deviation of complete operating condition velocity, $\mu_{6}$ is the weight factor of the standard deviation of the steering-wheel angle under complete working conditions, $\beta_{6}$ is the amplitude factor of the standard deviation of the steering-wheel angle under complete working conditions, $\varepsilon_{6}$ is the phase factor of the standard deviation of the steering-wheel angle under complete working conditions, ${\theta a}_{std}$ is the standard deviation of the steering-wheel angle under complete working conditions, $\mu_{7}$ is the maximum weight factor of the steering-wheel angle under complete working conditions, $\beta_{7}$ is the amplitude factor of the maximum steering-wheel angle under complete working conditions, $\varepsilon_{7}$ is the phase factor of the maximum steering-wheel angle under complete working conditions, and ${\theta a}_{max}$ is the maximum steering-wheel angle under complete working conditions. In this paper, select $\alpha_{all}=7.5,\;\mu_{5}=1,\;\beta_{5}=0.62,\;\varepsilon_{5}=-12.31,\;\mu_{6}=2,\;\beta_{6}=0.31,\;\varepsilon_{6}=-15.39,\;\mu_{7}=2,\;\beta_{7}=0.062,\;\varepsilon_{7}=-12.4.$

(3) $$G_{time}=\frac{\mu_{8}*arctan\left(\beta_{8}*T+\varepsilon_{8}\right)}{\pi }$$

where $G_{time}$ is the time score, $\mu_{8}$ is the time weight factor, $\beta_{8}$ is the time amplitude factor, $\varepsilon_{8}$ is the time phase factor, and T is the working condition completion time. In this paper, select $\mu_{8}=4,\;\beta_{8}=-0.04,\;\varepsilon_{8}=8.$

(4) $$G=G_{turn1}+G_{turn2}+G_{turn3}+G_{turn4}+G_{turn5}+G_{turn6}+G_{turn7}+G_{turn8}+G_{all}+G_{time}$$

where G is the score of the driving situation, $G_{turn1}\sim G_{turn8}$ is the score of turns 1~8, which is obtained by Formula (1) calculated, $G_{all}$ is the score of the complete working condition, and $G_{time}$ is the time score.

Combining Figure 10 reveals that B-level drivers have overall higher scores, with relatively small fluctuations compared to A-level drivers. Under the steer-by-wire system, the scores generally increase and stabilize over time, with scores in the later stages generally higher than those under the traditional steering system. Additionally, it can be observed that there are some differences in driving scores among drivers with different driving styles under the traditional steering system, but these differences are less pronounced under the steer-by-wire system. For A-level drivers, regardless of whether it is under the steer-by-wire system or the traditional steering system, the scores for individual driving sessions generally increase over time. Moreover, under the steer-by-wire system, A-level drivers tend to have higher scores overall compared to the traditional steering system. However, under the traditional steering system, A-level drivers experience larger fluctuations and differences in scores, possibly due to the instability of their driving behaviors. Furthermore, it can be observed that there are cases where A-level drivers exhibit more stable driving behaviors under the steer-by-wire system, but less stable behaviors under the traditional steering system.

Under the steer-by-wire system, A-level drivers gradually approach the level of B-level drivers after the tenth driving session, reaching a level similar to that of B-level drivers in the early stages of driving under the steer-by-wire system by the fifteenth session. However, under the traditional steering system, most A-level drivers still exhibit significant differences from B-level drivers even after ten sessions.

The adaptability of drivers to steer-by-wire systems is more pronounced in B-level drivers, while it is less evident in A-level drivers, with A-level drivers predominantly showing developmental changes. For B-level drivers, adaptability is primarily demonstrated in cornering. They make more corrections to the angle during cornering. However, after multiple drives, B-level drivers continuously adjust their control of the steering-wheel angle during cornering to achieve faster and better turning effects, relying on their driving experience and skills. The adaptability behavior of B-level drivers is mainly attributed to their habitual use of traditional steering systems. When the steering system changes, B-level drivers can transition their cornering techniques to adapt to the new system within a certain period. However, there is no situation where B-level drivers reject or fail to adapt to steer-by-wire systems. The multiple corrections observed in A-level drivers when driving with steer-by-wire systems are primarily due to their lack of driving experience and skills. This issue can be addressed through repeated and long-term driving to accumulate driving experience and skills, although the adaptability problem is not very pronounced in this group.

## 4. Discussion

### 4.1. Comparative Analysis of Different Types of Drivers Driving Different Steering Systems

Significant differences in speed and steering-wheel angle control exist between A-level and B-level drivers, regardless of whether traditional steering systems or steer-by-wire systems are employed.

Referring to Figure 4a and Figure 7, and combining the statistical calculations of average speeds from Table 3 and Table 4, notable disparities in speed between A-level and B-level drivers are evident across both traditional and steer-by-wire systems. Figure 4 illustrates that B-level drivers exhibit superior speed control, maintaining higher and more consistent speeds. In contrast, A-level drivers demonstrate poorer speed control, frequently maintaining lower speeds and struggling with stability. However, improvements are noted with increased driving experience.

Regarding Figure 7 and the statistical calculations of maximum absolute steering-wheel angles in Table 5 and Table 6, distinct differences between A-level and B-level drivers in various steering systems are observed. Figure 7b shows that differences in steering-wheel angle control between B-level and A-level drivers are less pronounced in traditional steering systems. Conversely, in steer-by-wire systems (Figure 7a), B-level drivers tend to have lower steering-wheel angles, albeit with more outliers. This variance may stem from differing operational principles and response modes between steer-by-wire and traditional systems. Consequently, B-level drivers may require an adjustment period to acclimate to the operational feel and response characteristics of steer-by-wire systems, potentially resulting in a less stable steering-wheel angle control when driving vehicles equipped with such systems.

Table 2 highlights that B-level drivers typically possess extensive driving experience and skills, enabling them to adeptly handle diverse driving situations and road conditions. They often exhibit familiarity with vehicle dynamics and handling techniques, which enhances their ability to manage complex driving scenarios. In contrast, A-level drivers, due to their limited experience, may react slower in emergencies or complex road conditions, resulting in less effective handling capabilities. A-level drivers require more practice and experience accumulation to enhance their proficiency and control levels compared to their B-level counterparts.

### 4.2. Changes in Driver Behavioral Adaptation Caused by Steer-by-Wire Systems

Referring to Figure 4a, Figure 7a and Figure 10a, A-level and B-level drivers demonstrate distinct adaptations in response to steer-by-wire systems. Specific adaptive changes are observed in Figure 5, Figure 6, Figure 8 and Figure 9.

B-level drivers, accustomed to traditional steering systems, must adapt to new driving experiences and operations when transitioning to steer-by-wire systems, particularly in steering-wheel angle control during turns (Figure 8 and Figure 9). However, changes in speed are less pronounced, as indicated in Figure 5 and Figure 6. Under steer-by-wire systems (Figure 8), B-level drivers tend to oversteer during low-speed turns and understeer during high-speed turns, necessitating more frequent adjustments to the steering-wheel angle. Despite this, their vehicle control is less stable compared to traditional systems. Nevertheless, leveraging their extensive driving experience and proficient skills, B-level drivers adeptly adjust steering-wheel angles, thereby mitigating instances of failed turns (Figure 8 and Figure 9).

Conversely, A-level drivers’ adaptability to steer-by-wire systems is relatively minor initially, focusing primarily on skill acquisition (Figure 4 and Figure 7). Their vehicle control is comparatively inferior to B-level drivers due to their limited driving experience. However, they exhibit comparable performance in traditional and steer-by-wire vehicles and may even demonstrate better steering-wheel angle control during turns under steer-by-wire systems (Figure 8). A-level drivers are in a learning phase with vehicle interaction, resulting in lower sensitivity to steering systems. Whether employing traditional or steer-by-wire systems, A-level drivers require learning opportunities, with adaptability challenges primarily centered on skill development.

As driving experience accumulates, behavioral adaptation for both A-level and B-level drivers undergoes gradual change, primarily influenced by varying levels of experience and skill (Figure 8 and Figure 9). The impact of driving style on adaptability is minimal, with habitual operations more influencing B-level drivers and skill learning more impacting A-level drivers. Over time, B-level drivers exhibit a more prominent adaptability to steering system changes, while A-level drivers gradually enhance the stability and accuracy of their driving behavior. Overall, the adaptability of the two types of drivers to the steer-by-wire system is positive, and the steer-by-wire system can give the driver a better driving experience.

### 4.3. Impact of Steer-by-Wire Systems on A-Level Drivers

As shown in Figure 4 and Figure 5 and Table 2, the impact of steer-by-wire systems on A-level drivers relative to traditional steering systems mainly manifests in turn control, speed perception, and driving skill learning. As shown in Figure 8 and Figure 9, in terms of turn control, under traditional steering systems, A-level drivers often experience instances of oversteering or understeering, requiring multiple corrections to the steering wheel. However, under steer-by-wire systems, although more corrections may be needed, overall instances of oversteering and understeering are fewer. The drivers grasp turning techniques more quickly and reduce the number of corrections, contributing to improved driving stability and safety.

As shown in Figure 5 and Figure 6, A-level drivers have a weaker perception of speed and cannot accurately judge vehicle speed through external scenery movement or dashboard readings. Under traditional steering systems, they often exhibit less control over speed, primarily using the method of releasing the accelerator pedal to decelerate. However, under steer-by-wire systems, as drivers find it easier to master steering techniques, their ability to control speed gradually improves. They begin to attempt more effective speed control by combining the brake pedal and accelerator pedal.

Steer-by-wire systems provide A-level drivers with a better driving experience and learning environment. By reducing the complexity of steering operations and providing more accurate steering feedback, A-level drivers find it easier to master turning techniques and adapt more quickly to various driving situations. Additionally, A-level drivers perceive steer-by-wire systems as more helpful for correcting turns, further strengthening their positive acceptance and motivation to learn this technology. This helps them to master driving skills more quickly, reduce traffic accidents, and enhance driving safety and stability.

## 5. Conclusions

As an advanced vehicle control technology, steer-by-wire systems have various impacts on driver behavioral adaptability. Compared to traditional mechanical steering systems, steer-by-wire systems offer higher feedback precision and sensitivity, which may require drivers to take some time to adapt to the more responsive steering feel and more accurate steering feedback. Additionally, steer-by-wire systems feature adjustable steering force and angle functions, necessitating driver adaptation to different settings for optimal driving comfort and maneuverability. Moreover, steer-by-wire systems provide more precise and smoother steering control, potentially prompting drivers to gradually change their driving behaviors and habits, such as performing steering operations more frequently or preferring smaller steering angles. Furthermore, drivers need to adapt to the faster and more precise steering response, as well as higher steering reaction speed and driving decision-making capability offered by steer-by-wire systems. The use of steer-by-wire systems may also affect drivers’ driving skills and training levels, as they may gradually develop driving skills more suited to this advanced technology, including a more proficient operation of steering systems and more flexible handling of various driving situations.

Through the above driving-simulator experiment and questionnaire results, when discussing the influence of the steer-by-wire system on driver-behavior adaptability, we find that the influence of the steer-by-wire system on driver-behavior adaptability is multi-dimensional. According to the experiment results of this paper, it can be seen that experienced drivers (corresponding to the B-level drivers above) and novice drivers (corresponding to the A-level drivers above) show different adaptability characteristics. Experienced drivers are more susceptible to the impact of inertial operation, while novice drivers are more susceptible to the impact of skill learning. In terms of turn control, speed perception, and driving skill learning, the impact of steer-by-wire systems on novice drivers manifests as a better driving experience and learning environment, making it easier for them to master driving skills by reducing the complexity of steering operations and providing more accurate feedback. Factors influencing driver adaptability to steer-by-wire systems involve the traditional steering vehicle driving experience, driving skills, driving style, trust, acceptance, and understanding of steer-by-wire systems. To facilitate drivers’ faster adaptation to steer-by-wire systems, it is necessary to comprehensively consider these factors and provide effective guidance, as well as clear and accurate system feedback and personalized adjustment functions.

The limited sample size and the use of a driving simulator may restrict the general applicability of our findings. Considering the pilot nature of this study, caution must be exercised when extrapolating the results to a broader population. Therefore, we recognize the necessity of conducting more extensive research involving a larger and more diverse driver sample. In future research, we will employ high-fidelity driving simulators and real-world vehicle tests to minimize the impact of simulator-specific biases on the results. Additionally, we will consider a wider range of traffic environments and road conditions in the experiments. Moreover, we will carefully examine factors that may influence test outcomes, such as system delay and other potential variables.

In future studies, we will delve deeper into the effects of driving style, driving skills, gender, age, personality traits, and system familiarity on adaptability. We plan to further refine driver classification and explore the differences in driver adaptability to steer-by-wire systems more comprehensively. By increasing the frequency of data collection and extending observation periods, we aim to gain a more thorough understanding of how driver behavior evolves with prolonged use and how long-term exposure to these systems affects driver comfort, performance, and adaptability. These insights will provide more comprehensive guidance for the future design and optimization of steer-by-wire systems.

## Figures and Tables

**Table 1:** Feature parameters of each drive for A-level and B-level drivers.

	**A-Level Driver**							
	**Steer-by-Wire System**				**Traditional Steering System**			
	**1**	**2**	**…**	**15**	**1**	**2**	**…**	**15**
Average Vehicle Speed (km/h)	52.64	57.07	…	55.91	53.45	57.91	…	56.79
Longitudinal Speed Standard Deviation	24.89	22.74	…	21.18	16.37	18.98	…	19.14
Maximum Steering-Wheel Angle (°)	87.08	97.18	…	95.64	228.02	187.45	…	174.87
Steering-Wheel Angle Standard Deviation	29.29	30.61	…	30.12	43.77	43.04	…	42.84
Driving Completion Time (s)	86.00	92.84	…	95.32	103.38	95.50	…	96.28
	**B-Level Driver**							
	**Steer-by-Wire System**				**Traditional Steering System**			
	**1**	**2**	**…**	**15**	**1**	**2**	**…**	**15**
Average Vehicle Speed (km/h)	45.43	42.85	…	40.97	34.15	48.04	…	42.90
Longitudinal Speed Standard Deviation	14.04	13.83	…	13.15	13.23	11.75	…	12.27
Maximum Steering-Wheel Angle (°)	203.17	103.03	…	95.38	232.10	223.30	…	157.60
Steering-Wheel Angle Standard Deviation	35.42	25.91	…	24.90	49.99	45.53	…	40.77
Driving Completion Time (s)	115.84	124.38	…	129.94	155.61	114.38	…	124.45

In the table, 1, 2 indicates the number of times the driver drove. Since the tests are repeated many times for each driver, this table represents only the first three tests under each steering system.

**Table 2:** Summary of the questionnaire survey results.

	A_1_	A_2_	…	A_7_	B_1_	B_2_	…	B_7_
Driving style (Careful, General, Aggressive)	General	General	…	Aggressive	General	Aggressive	…	Careful
driving skill (Excellent, Good, Medium, Pass, Fail)	Good	Medium	…	Medium	Excellent	Good	…	Good
Understanding of the steer-by-wire system (Very familiar, Familiar, Uncertain, Unfamiliar, Very unfamiliar)	Familiar	Unfamiliar	…	Unfamiliar	Familiar	Familiar	…	Familiar
Trust in the steer-by-wire system (Very trust, Trust, Uncertain, Distrust, Very distrust)	Trust	Trust	…	Uncertain	Trust	Trust	…	Uncertain
Acceptance of steer-by-wire system (Very acceptable, Acceptable, Uncertain, Unacceptable, Very unacceptable)	Acceptable	Uncertain	…	Unacceptable	Acceptable	Acceptable	…	Acceptable
Steer-by-wire system driving experience (Very good, Good, Uncertain, Bad, Very bad)	Good	Good	…	Uncertain	Good	Good	…	Good
Feel the difference between the two types of steering systems	Yes	Deny	…	Deny	Yes	Yes	…	Yes

In the table, A_m_ (m = 1, 2, 3, 4, 5, 6, 7) represents the mth A-level driver, and B_m_ (m = 1, 2, 3, 4, 5, 6, 7) represents the mth B-level driver. This notation will be used throughout the paper to represent A-level and B-level drivers.

**Table 3:** Statistics of average vehicle speed for drivers under the steer-by-wire system.

	**A_1__SBW**	**A_2__SBW**	**A_3__SBW**	**A_4__SBW**	**A_5__SBW**	**A_6__SBW**	**A_7__SBW**	**A_SBW**
Mean	41.901	46.806	37.529	53.385	50.106	45.480	43.423	45.519
Median	42.226	45.640	37.318	53.364	50.836	44.843	43.645	44.749
Range	10.984	17.225	7.953	9.330	9.387	21.071	8.010	23.672
Standard deviation	2.929	4.472	2.086	2.456	2.550	5.201	2.308	5.866
	**B_1__SBW**	**B_2__SBW**	**B_3__SBW**	**B_4__SBW**	**B_5__SBW**	**B_6__SBW**	**B_7__SBW**	**B_SBW**
Mean	59.137	62.960	52.234	57.750	56.262	52.808	48.008	55.594
Median	59.547	63.302	51.436	58.531	56.020	52.204	47.867	56.031
Range	9.255	7.626	11.291	7.577	11.811	8.326	4.909	20.642
Standard deviation	2.462	1.954	2.799	2.071	3.196	2.714	1.607	5.205

In the table, A_m__SBW (m = 1, 2, 3, 4, 5, 6, 7) represents the data of the mth A-level driver driving under the steer-by-wire system; B_m__SBW (m = 1, 2, 3, 4, 5, 6, 7) represents the data of the mth B-level driver driving under the steer-by-wire system; A_SBW represents all A-level drivers’ data driving under the steer-by-wire system; B_SBW represents all B-level drivers’ data driving under the steer-by-wire system. The mean is represented by M, the median is represented by Med, the range is represented by R, and the standard deviation is represented by SD. The same representation applies in the following text.

**Table 4:** Statistics of average vehicle speed for drivers under the traditional steering system.

	**A_1__nSBW**	**A_2__nSBW**	**A_3__nSBW**	**A_4__nSBW**	**A_5__nSBW**	**A_6__nSBW**	**A_7__nSBW**	**A_nSBW**
Mean	41.067	44.792	41.569	44.423	54.719	41.688	55.142	46.200
Median	40.466	45.158	40.965	45.373	54.329	41.110	54.476	45.373
Range	13.897	6.658	6.819	15.901	11.241	19.199	15.700	29.864
Standard deviation	3.543	2.123	2.201	4.309	2.880	5.673	3.806	6.740
	**B_1__nSBW**	**B_2__nSBW**	**B_3__nSBW**	**B_4__nSBW**	**B_5__nSBW**	**B_6__nSBW**	**B_7__nSBW**	**B_nSBW**
Mean	58.894	57.179	52.269	57.888	56.914	58.192	56.189	56.789
Median	59.413	57.350	52.179	56.836	58.869	57.318	56.072	56.986
Range	8.174	15.283	9.334	6.944	11.443	9.605	9.024	15.394
Standard deviation	2.046	4.857	2.832	2.212	3.672	3.225	2.502	3.695

In the table, A_m__nSBW (m = 1, 2, 3, 4, 5, 6, 7) represents the data of the mth A-level driver driving under the traditional steering system; B_m__nSBW (m = 1, 2, 3, 4, 5, 6, 7) represents the data of the mth B-level driver driving under the traditional steering system; A_nSBW represents all A-level drivers driving under the traditional steering system; B_nSBW represents all B-level drivers driving under the traditional steering system. The same representation applies in the following text.

**Table 5:** Statistics of maximum absolute steering-wheel angle for drivers under the steer-by-wire system.

	**A_1__SBW**	**A_2__SBW**	**A_3__SBW**	**A_4__SBW**	**A_5__SBW**	**A_6__SBW**	**A_7__SBW**	**A_SBW**
Mean	115.96	138.89	85.76	203.93	169.15	154.03	137.20	143.56
Median	106.56	121.21	84.32	218.26	185.00	147.11	123.76	129.15
Range	113.18	141.81	76.54	98.18	162.65	125.93	134.60	187.17
Standard deviation	29.05	45.99	17.95	29.02	51.52	43.59	39.62	51.15
	**B_1__SBW**	**B_2__SBW**	**B_3__SBW**	**B_4__SBW**	**B_5__SBW**	**B_6__SBW**	**B_7__SBW**	**B_SBW**
Mean	94.38	143.04	87.93	97.83	95.24	132.81	145.58	113.83
Median	92.17	144.00	83.42	98.49	94.64	129.22	146.66	108.94
Range	47.03	83.57	73.11	38.38	62.11	68.67	67.85	115.32
Standard deviation	11.13	21.92	19.30	14.14	17.06	21.45	20.60	29.58

**Table 6:** Statistics of maximum absolute steering-wheel angle for drivers under the traditional steering system.

	**A_1__nSBW**	**A_2__nSBW**	**A_3__nSBW**	**A_4__nSBW**	**A_5__nSBW**	**A_6__nSBW**	**A_7__nSBW**	**A_nSBW**
Mean	157.18	162.54	158.53	225.15	159.87	199.28	248.27	187.26
Median	145.77	159.36	157.12	238.03	154.13	200.48	243.96	173.08
Range	106.14	95.89	51.70	166.90	87.94	151.07	91.60	176.72
Standard deviation	31.24	23.61	16.21	48.30	27.02	40.34	22.49	46.39
	**B_1__nSBW**	**B_2__nSBW**	**B_3__nSBW**	**B_4__nSBW**	**B_5__nSBW**	**B_6__nSBW**	**B_7__nSBW**	**B_nSBW**
Mean	144.46	179.06	138.09	135.18	177.95	210.68	133.00	159.77
Median	140.60	188.22	146.96	137.24	174.93	208.40	125.37	158.49
Range	60.08	107.44	90.11	54.40	58.14	100.57	74.80	162.89
Standard deviation	15.97	29.12	32.56	17.44	15.71	27.20	23.87	36.18

## Data Availability

Data are contained within the article.
